# A novel mechanism for regulation of the type I IFN response by herpesvirus deconjugases

**DOI:** 10.15698/mic2018.05.633

**Published:** 2018-04-11

**Authors:** Soham Gupta, Päivi Ylä-Anttila, Maria G. Masucci

**Affiliations:** 1 Department of Cell and Molecular Biology, Karolinska Institutet, S-17177 Stockholm, Sweden.

**Keywords:** Epstein-Barr virus, Herpesvirus, deconjugase, RIG-I signalosome, type I IFN

## Abstract

Upon infection, viral nucleic acids are recognized by germline-encoded pattern-recognition receptors (PRRs), and cytosolic retinoic acid-inducible gene I (RIG-I)-like helicases (RLHs) that initiate signaling pathways resulting in the production of type I IFN and pro-inflammatory cytokines. Binding of RIG-I to viral nucleic acids triggers the formation of the RIG-I signalosome where RIG-I is ubiquitinated by the TRIM25 ligase and, with the help of 14-3-3 scaffolds, further translocated to mitochondrial anti-viral signalling proteins (MAVS). Subsequent ubiquitination-mediated events trigger transcriptional activation of the effectors of innate immunity. We have found a new mechanism by which herpesviruses interfere with this signalling pathway to favour the establishment of latency and promote virus replication. The cysteine protease encoded in the conserved N-terminal domain of the herpesvirus large tegument protein binds to 14-3-3 proteins and forms a tri-molecular complex with TRIM25, promoting the activation and autoubiquitination of the ligase. RIG-I is recruited to the complex but its ubiquitination is drastically reduced, which effectively inactivates downstream signalling and blocks the type I IFN response.

Post-translational modification of proteins by covalent attachment of small polypeptides such as ubiquitin (Ub) and ubiquitin-like molecules controls a multitude of cellular functions by regulating protein turnover, their localization, interactions and functions. Ubiquitination is mediated by an enzymatic cascade that comprises the ATP-dependent Ub-activating enzyme, a conjugating enzyme and a substrate-specific ligase that transfers Ub to the acceptor protein. The reaction is reversed by deconjugases that hydrolyze the covalent bond formed between Ub and the substrate. Viruses interfere with ubiquitination in two major ways. Many viruses encode proteins that redirect the activity of the conjugation machinery towards new substrates whose modification favors infection. In addition, some viruses encode functional homologs of ligases and deconjugases, as exemplified by the conserved family of herpesvirus deconjugases. The viral enzymes are encoded in N-terminal domain of the major tegument protein that is produced during the early and late phases of the productive virus cycle and subsequently incorporated into virus particles. While important roles of the enzyme in the regulation of virus production, infectivity and antiviral response have been elucidated the cellular and viral substrates remain largely unknown.

We used a co-immunoprecipitation and mass spectrometry approach to search for interacting proteins and substrates of BPLF1, the deconjugase encoded by the human oncogenic herpesvirus Epstein-Barr virus (EBV). Several members of the 14-3-3-family of molecular scaffold proteins were identified as putative BPLF1 binding partners. The 14-3-3 proteins are conserved regulatory molecules expressed in all eukaryotic cells that control the activity of a multitude of signaling pathways. By comparing the BPLF1 and 14-3-3 interactomes we have identified the TRIM25 ligase as a shared interacting partner. 14-3-3 and TRIM25 are essential components of the RIG-I signalosome where 14-3-3 stabilizes the interaction of TRIM25 with RIG-I and facilitates RIG-I ubiquitination and the translocation of the active complex to MAVS for downstream signaling. Both catalytically active and inactive BPLF1 can form stable tri-molecular complexes with 14-3-3 and TRIM25, but in the presence of catalytically active BPLF1 mono and di-ubiquitinated TRIM25 species were regularly detected. This is likely to be a consequence of the BPLF1-dependent for-mation of TRIM25 oligomers that activate the ligase by allowing correct positioning of the substrate and Ub-loaded conjugating enzyme. One interesting question is why the modification of TRIM25 is not observed in the presence of catalytically inactive BPLF1 that retains the capacity to bind 14-3-3. One possible explanation is that the formation of stable TRIM25 oligomers is dependent on deubiquitination of 14-3-3 or a yet unidentified component of the complex. The capacity of active BPLF1 to stabilize TRIM25 by inhibiting ubiquitination by a different cellular ligase, such as the linear ubiquitin ligase assembly complex (LUBAC), could also play a role in promoting oligomerization. Alternatively, the formation of BPLF1:14-3-3:TRIM25 tri-molecular complexes may be sufficient to promote the polyubiquitination of TRIM25 but long chains may be trimmed down to mono- or di-ubiquitinated species by the catalytically active BPLF1. If so, polyubiquitinated TRIM25 may accumulate in the presence of catalytically inactive BPLF1. Further experiments will be required to discriminate between these possibilities.

In spite of the capacity of BLPF1 to promote the activation of TRIM25, expression of the viral enzyme was associated with failure to detect RIG-I ubiquitination following triggering of the signaling pathway by treatment with poly(I:C) or expression of a constitutively active RIG-I. Two possible scenarios may explain this observation. Recruitment of the viral deconjugase to the 14-3-3:TRIM25:RIG-I complex may directly counteract the activity of TRIM25 and promote the release of unmodified RIG-I. Alternatively, the presence of mono or di-ubiquitinated TRIM25 may weaken the interaction of TRIM25 with RIG-I causing the release of ubiquitinated RIG-I and its subsequent deubiquitination by cellular deconjugase or unbound BPLF1. In either case, the deubiquitination of RIG-I and its release from the signalosome complex interrupts the signaling cascade leading to inhibition of the type I IFN response. These findings illustrate a previously unrecognized mechanism by which herpesviruses may counteract the activation of innate anti-viral responses (Figure 1).

**Figure 1:**
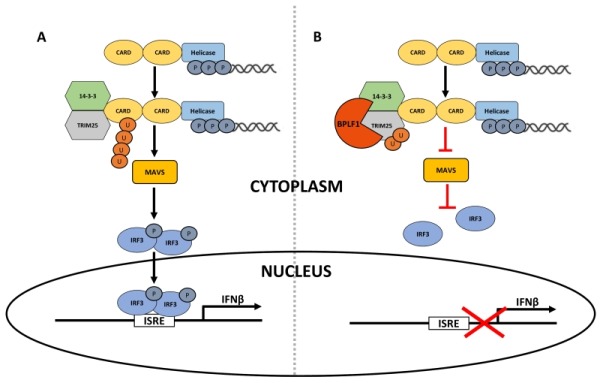
FIGURE 1: Proposed model for inhibition of the RIG-I signalosome by herpesvirus deconjugases. **(A) **Following binding to nucleic acids RIG-I undergoes a conformational change that exposes the CARD domains. Activated RIG-I binds to TRIM25, which promotes ubiquitination of the first CARD domain. 14-3-3 stabilizes the RIG-I-TRIM25 complex and promotes its translocation to MAVS where subsequent ubiquitin-regulated events lead to phosphorylation of the IRF3 transcription factor (p-IRF3). Activated IRF3 is translocated to the nucleus where it binds to IFN-response element (ISRE) leading to the activation of type I IFN response. Activation events are indicated by black arrows. **(B) **BPLF1 and the homologs encoded by other herpesviruses form a tri-molecular complex with 14-3-3 and TRIM25, which leads to activation and autoubiquitination of the ligases. A yet uncharacterized sequence of events results in failure to ubiquitinate RIG-I and functional inactivation of the RIG-I signalosome. Inhibitory events are indicated by red inverted T.

While the capacity of herpesvirus deconjugases to interfere with various components and different steps of the IFN response was reported before, we found that the capacity to bind to and regulate the activity of the 14-3-3:TRIM25 complex and inhibit RIG-I ubiquitination is shared by the BPLF1 homologs encoded by other herpesviruses, implying that this early step of the activation cascade may be the primary target of these viral enzymes. Interestingly, we noticed weaker binding to 14-3-3 and consequently weaker TRIM25 mono-ubiquitination and stronger RIG-I ubiquitination in cells expressing the HSV1-UL36, suggesting that interference with the activity of 14-3-3 may not be critical for the lifecycle of this virus.

Our findings shed an interesting new light on the pathophysiology of herpesvirus infection and the strategies used by the virus to bypass cellular and immune control mechanisms. The large tegument proteins of herpesviruses are expressed during virus replication and are packaged into virus particles for delivery to newly infected cells Thus, their enzymatic activity may play a double role in the virus life cycle by extending the time of unchecked productive infection, and by participating in the cellular reprogramming that allows the establishment of latent infections. A precise mapping of molecular interactions involved in the functional inactivation of the RIG-I signalosome will be required to guide the development of small molecule inhibitors that could effectively potentiate the antiviral response and decrease or event to even prevent the establishment of persistent infections.

